# On Seclusion, as Employed at Hanwell in the Treatment of the Insane

**Published:** 1841-04

**Authors:** 


### ON SECLUSION, AS EMPLOYED AT HANWELL IN THE TREATMENT OF THE INSANE.

[Among the numerous misrepresentations that have gone abroad respecting the admirable improvements introduced into Hanwell by Dr. Conolly, there has been none greater than that which has converted the temporary and occasional seclusion of violent patients into an habitual system of restraint and punishment as severe and objectionable as the practices he has abolished. In order to expose the utter inaccuracy of such statements, we lay before our readers the section on Seclusion, from the Report recently presented by Dr. Conolly to the county magistrates; and we can testify from personal knowledge of the proceedings at Hanwell, that the practice of the Asylum is in strict accordance with the principles there laid down.]

*Seclusion.* All the substitutes for restraint are, like restraint itself, liable to be abused; but none can be made such instruments of cruelty by abuse. All are also liable to great misrepresentation: and none more so than that which is of all the most useful, the most simple, and the most approved of by the highest medical authorities; namely, seclusion. By seclusion is meant, temporary protection of the maniac from the ordinary stimuli acting upon the senses in the refractory wards of a lunatic asylum. He is abstracted from noise; from the spectacle of a crowd of lunatics; from meeting those who are almost as violent as himself; and from every object likely to add to his irritation. But the mode in which seclusion is effected is also important to securing the benefits of it. If resorted to with violence, if accompanied with expressions of anger or contempt, if stigmatised as a punisment, and if followed by neglect, it may produce all the evil moral effects of restraint itself. If injudiciously persevered in in very recent cases, it exasperates instead of calming. The patient requires freedom of action; is relieved by strong muscular exercise; and this should be provided for by such a subdivision of airing courts as would leave one for the occasional use of a single patient, at least for a few hours in the day. After being indulged in active voluntary exercise for an hour, two hours, or such period as may seem desirable, the patient should be secluded. Calmness and sleep will sometimes follow; or sufficient tranquillity to enable the attendants and officers to talk to the patients with effect.

Under the system of restraints, when a patient became noisy and violent, and particularly when some mischief had been committed by him, it was considered necessary, and it was the usual practice, to overpower him, and to put him in some kind of strait-waistcoat. This was done with great difficulty, and with much danger to the attendants. Observation has convinced the resident physician that this was a useless, and even hurtful, mode of management. It was like endeavouring to smother a fierce fire by heaping very combustible meterials upon it. A maniac in the midst of his paroxysm, like a man in a violent fit of passion, should be interfered with as little as possible. The violence, which if met by violence will become still more aggravated, will often, if left to itself, subside even in the course of five or ten minutes. Whatever the duration of the violent accession, its continuance is a bar to anything but such management as protects the patient and those about him. It is in intervals of calmness that the foundations of moral treatment must be laid, and the confidence of the patient gained. To acquire this confidence is the key-stone of all moral treatment; and nothing will so much oppose its acquisition as brutal or even impatient usage during the paroxysm.

In the meantime, supposing the violent state to continue, the other patients should be removed from the neighbourhood of the one who is excited; all obvious means of mischief should be guarded against; and the attendants, although not directly interfering, should be watchful and ready. A soothing word now and then, or something new to attract the attention, may be admissible; but all with discretion. Supposing, as must often happen, that the violent fit does not immediately subside, and that the patient continues to vociferate, to swear, to abuse and threaten those about him, and to endeavour to strike them, something more must be done. But in this condition the worst thing that can be done is to struggle violently with him, to overpower him, and to mortify him by putting on bodily restraint. The only reasonable object of any treatment is to tranquillize the violent man: and of all modes of effecting this, surely violence is the most unreasonable. Seclusion effects the object more certainly than restraint, and without any violence at all. A lunatic is seldom, even in his most raving fits, insensible to what is said to him: he will often show, among his wildest and most extravagant expressions, that he is watchful of the conduct of those about him; and when the ordinary observer would expect nothing from him but what indicated savage fury, those who are patient with him, and who, regardless of his wildness, continue to indicate their kind feelings towards him, will find that sometimes his voice falters, and his eyes fill with tears. These symptoms of emotion are very transient; but they show that the sensibilities are not quite suppressed; and they warn the practitioner, in language that ought not to be disregarded, to abstain from everything which can further wound or oppress the feelings of an almost ruined mind. These circumstances are now mentioned as bearing upon the manner in which the seclusion of a violent patient should be effected. Three or four attendants, possessed of courage and good temper, should surround him; and telling him that he would be much better if quiet, and in his own room, should endeavour, by gentle occasional efforts, to induce him to walk into it. It will sometimes be found, that although he protests loudly against the measure, his steps gradually proceed in the direction required. At the same time, steadiness and strength may be required to prevent his retrograding; but well-qualified attendants will not on this account resort to violence. If he strikes or kicks them, they must of course effect their purpose as speedily as possible, and with steadiness, and even with force; but always without passion. As soon as the patient is thus placed in his room, he is not unfrequently found to become quiet; or if he continues to talk loudly, it is not for a long period. In all probability he will soon lie down on his bed, and go to sleep. If he continues violent, he is at all events out of harm's way. He will very seldom attempt to hurt himself; and he can hurt no one else. The window of his room should in all cases be secured by an efficient shutter and lock. The bedstead, which should be of wood, should be fastened to the floor, and remote from the window. Sufficient light should be admitted through holes made in the window-shutter to enable the attendants, by looking through the inspection plate in the door, frequently to ascertain the state of the patient.

The abuse to which this seclusion is liable is that of being too prolonged. A troublesome patient being once locked up, it is natural that the attendants should have no very anxious desire to let him out: but if the superintendent is assisted by officers of proper activity, this abuse should be impossible. The seclusion should in all cases be immediately reported; and after two or three hours, some officer of the asylum should visit the patient, or at least look at him through the inspection-plate, so as to judge of the propriety of the seclusion being continued or put an end to. This should be done with great circumspection. If even the cover of the inspection-plate is moved roughly and noisily, the patient is roused and irritated. Still greater mischief may be done by prematurely going into his room. A daily report should be made to the superintendent of the patients who have been in seclusion, and the number of hours they have been secluded. It is impossible to lay down rules for the general length of seclusion. Three hours, two hours, or one hour, in many cases answer every purpose. In other instances, after four or five hours, although the patient should be brought out, and a trial given of his capacity to behave well, it is not found practicable to have him at large among the other patients; and it is better to keep him in his room till the next day. Many patients liable to periodical excitement, and especially females, are far more comfortable if kept in seclusion during the whole period of their excitement.

When the female patients are put in seclusion in consequence of any sudden outburst of passion, a very usual effect upon them is a vehement fit of crying and sobbing. In such cases, after a short interval, it is desirable that the patient should be talked to and soothed; and the seclusion should not be prolonged. If such a patient is neglected, seclusion will only produce sullenness. There are other cases in which a female patient, who would strike and kick those about her if at large, will become instantly quiet when put in seclusion; and, when seen through the inspection-plate, will seem so extremely tranquil, as to make it appear to those unacquainted with her, that she might be suffered to be at large without inconvenience: but if the door is incautiously opened, such a patient will be found to spring suddenly upon the intruder. The character of such patients becomes familiar to the attendants; but from perverseness, or obstinacy, they sometimes thwart the intention of the superintendent by letting out the patient to astonish a visitor, or by prolonging the seclusion when they know it to be no longer necessary.

In very young female patients, disposed to occasional violence, seclusion requires to be applied with peculiar precaution. The darkness appears to alarm them; and the solitude seems to aggravate the paroxysm. In all respects these patients require particular attention; the greater number of them being curable; yet so strongly affected, favorably or unfavorably, by all the circumstances around them, as easily to be rendered incurable by neglect or mismanagement.

There are many patients subject to paroxysms of excitement of about a week's duration, who, of their own accord, will keep in their rooms at such a time; and who, although the door is not locked, will seldom offer to come out. There were no patients more injured by the imposition of restraint than these: the character of some of them, even during their most excited state, is improved since its discontinuance; and, at other times, instead of being a terror to the attendants and the officers, they are among the most affectionate and grateful patients in the house.

The resident physician dwells more minutely on seclusion, because he considers it as one of the most important of curative means, and as one of the least objectionable substitutes for every kind of restraint. It is open to no objection which is not doubly applicable to restraint. All the possible evils of seclusion were included among the innumerable evils of bodily coercion. While the patients who were permitted to walk about in restraint were still capable of inflicting injury upon others, they were not protected from causes of irritation, or from the attacks of other patients. When put in seclusion, it was a seclusion which did not tranquillize. The arms or the hands were closely confined to the body; or the arms, or the legs, were strapped or chained to the bedstead; or the head was confined by a strap round the neck. In this state they were left for days or for weeks, in the most miserable condition in which a human being could be placed; and often to the total ruin of all habits of cleanliness. The patients themselves, who now come to us from other asylums, reported, "violent and dirty," sometimes remark, that they could not be otherwise than dirty when they are chained down in a deep bed like a trough. The same patients, being freed from all restraint the moment they arrive at Hanwell, seldom prove dirty, and not always violent.

To obviate every objection to seclusion, all the resources of the non-restraint system must be brought to bear upon it. The destruction of bedding and of clothing should be prevented by bedding properly secured in ticking covers, strongly sewed; and by clothing of the same material, fastened by small locks instead of buttons. If the patient will not lie in bed, warm boots, similarly fastened, should be constantly worn. So important do even trifling matters become as auxiliaries to this kind of treatment, that it may be right to mention, that the ticking should be of the best and strongest manufacture, and carefully sewed with the strongest thread; or, in the case of male patients, with twine. Without proper precautions of this kind, the attendants will very probably report that the dresses and blanket-cases are useless. The attendants should be continually exhorted to watch for all favorable opportunities to get the patient out of his room and into the open air: and the cleanliness of his apartment and person, and the proper administration of food to him, should be most scrupulously observed.

All proper medical means are compatible with this treatment: and although some cases must be expected to be much more troublesome than others; although, indeed, it is known to all familiar with insanity, that there are cases, in which more or less of maniacal excitement will continue for six, eight, or ten months; yet under this treatment, the management of such cases will be found less distressing; the temper and habits of the patients more controllable; and the return to reason steadier, and made with more gratifying circumstances, than where the confidence of the patient has been shaken, and the excitement of the malady aggravated, by violence of any kind whatever. From the statements then made by the recovering patients, the superintendent will learn, and having learned, ought never to forget, that every act of violence, that every word of irritation, that every injudicious expression, of which the attendants were guilty, or into which he himself was betrayed, during the most excited period of the patient's malady, remains recorded in the patient's mind; and that no act or word of kindness, no remission of severity, no little indulgence, no encouragement held out to the poor sufferer, passed unregarded.

---

